# Rab8 and Rabin8-Mediated Tumor Formation by Hyperactivated EGFR Signaling via FGFR Signaling

**DOI:** 10.3390/ijms21207770

**Published:** 2020-10-20

**Authors:** Junghwa Choi, Jee Young Sung, Saerom Lee, Jungyoen Yoo, Christopher Rongo, Yong-Nyun Kim, Jaegal Shim

**Affiliations:** 1 Research Institute of National Cancer Center, 323 Ilsan-ro, Goyang, Gyeonggi-do 10408, Korea; jhchoi425@gmail.com (J.C.); sungjy@ncc.re.kr (J.Y.S.); 74419@ncc.re.kr (S.L.); dh34111@naver.com (J.Y.); 2 Waksman Institute of Microbiology, Rutgers, The State University of New Jersey, 190 Frelinghuysen Road, Piscataway, NJ 08854, USA; crongo@waksman.rutgers.edu

**Keywords:** EGFR, FGFR, Rab8, Rabin8, cancer

## Abstract

The epidermal growth factor receptor (EGFR) signaling is important for normal development, such as vulval development in *Caenorhabditis elegans*, and hyperactivation of the EGFR is often associated with cancer development. Our previous report demonstrated the multivulva (Muv) phenotype, a tumor model in *C. elegans* (*jgIs25* strain) by engineering LET-23/EGFR with a TKI-resistant human EGFR T790-L858 mutant. Because Rab proteins regulate vesicle transport, which is important for receptor signaling, we screened the RNAi in the *jgIs25* strain to find the Rabs critical for Muv formation. Herein, we show that *rab-8* RNAi and the *rab-8 (-/-)* mutation effectively reduce Muv formation. We demonstrate that RABN-8, an ortholog of Rabin8, known as a GEF for Rab8, is also required for Muv formation by promoting the secretion of EGL-17/FGF from vulval precursor cells. In addition, FGFR inhibitors decreased Muv formation mediated by mutant EGFR. Our data suggest that Rab8 and Rabin8 mediate Muv formation through FGF secretion in the EGFR-TKI-resistant nematode model. Furthermore, FGFR-TKIs more effectively inhibit the growth of lung cancer cell lines in H1975 (EGFR T790M-L858R; EGFR-TKI-resistant) than H522 (wild-type EGFR) and H1650 (EGFR exon 19 deletion; EGFR-TKI-sensitive) cells, suggesting that FGFR-TKIs could be used to control cancers with EGFR-TKI-resistant mutations.

## 1. Introduction

The epidermal growth factor receptor (EGFR) is a major oncogene targeted by anticancer reagents, and many EGFR-tyrosine kinase inhibitors (TKIs) are now widely used in cancer treatment. First-generation EGFR-TKIs, such as gefitinib and erlotinib are effective against tumors with activated mutant forms of the EGFR, including a 19th exon deletion or an L858R mutation [1,2]. However, most cancer patients treated with these drugs exhibit EGFR-TKI resistance within 9–14 months [3]. The numerous causes of EGFR-TKI resistance include secondary mutations in the EGFR, activation of alternative signaling pathways, aberrance of the downstream pathways, and impairment of the EGFR-TKI-mediated apoptosis pathway [4]. Among secondary EGFR mutations that confer resistance, the EGFR T790M mutation is the most important because it accounts for 50% of the total EGFR-TKI-resistant mechanisms [5]. One approach to overcome resistance due to EGFR T790M has been the development of new EGFR targeting drugs, including osimertinib (Tagrisso) [6]. However, osimertinib treatment can also lead to drug resistance through new EGFR mutations and other unknown mechanisms, highlighting the need for new approaches [7].

EGFR is known to crosstalk with various receptor systems including integrins and platelet-derived growth factor receptor (PDGFR), and thus contributes to cancer malignancy [8]. The fibroblast growth factor receptor (FGFR) is suggested to have crosstalk with the EGFR in cancer cells, such as breast cancer as well as normal development such as skeletogenesis [9,10]. Overexpression of the FGFR is a cause of anticancer drug resistance, and EGFR-TKI treatment upregulates FGFR signaling in lung adenocarcinoma cell lines [11]. Indeed, dual inhibition of the EGFR and the FGFR is effective in non-small cell lung cancer and breast cancer [12]. Long-term cancer treatment requires a better understanding of resistance mechanisms and pathway crosstalk combined with novel anticancer drugs targeting these mechanisms.

Vesicle trafficking supports epithelial cell polarity, and its dysregulation is associated with major cancer events such as carcinogenesis, invasion and metastasis [13]. Vesicle trafficking can transfer growth factor receptors directly to the cell surface or can eliminate them through endocytosis. On the other hand, it is also possible to regulate the signal transduction of growth factor receptors by regulating ligand secretion. The small GTPase Ras-associated binding (Rab) proteins are key regulators of vesicle trafficking, with more than 70 Rab members regulating diverse routes of vesicle transport in humans [14]. The endocytic pathway of the EGFR is well known to be regulated by several Rabs, including Rab5, Rab7 and Rab11. Rab5 promotes EGFR endocytosis from the plasma membrane to early endosomes, whereas Rab11 mediates recycling of EGFRs from recycling endosomes to the plasma membrane. Meanwhile, Rab7 is required for EGFR transport to lysosomes for degradation [15]. In breast cancer cells, insulin-like growth factor II secretion by Rab27A promotes invasion and metastasis potential [16]. Such regulators of growth factor receptor trafficking and ligand secretion are potential targets for cancer therapeutic approaches.

*Caenorhabditis elegans* vulval development provides a genetic model system for studying signal transduction, as both the EGFR and FGFR signaling pathways are required for normal vulval development and its function in laying eggs [17,18]. Epidermal growth factor (EGF) is a major induction signal for vulval precursor cell (VPC) proliferation and differentiation [19]. LET-23, the *C. elegans* EGFR ortholog, acts in the VPCs to transduce the inductive signal required for differentiation of the vulval epithelia. Reduction-of-function (*rf*) mutations in the EGFR result in a vulvaless (Vul) phenotype, whereas gain-of-function (*gf*) mutations result in the formation of additional vulva tissue, which is known as the multivulva (Muv) phenotype [17,20]. We previously generated a tumor model exhibiting the Muv phenotype in *C. elegans* by introducing the human EGFR mutants EGFR L858R and EGFR T790M-L858R into the *C. elegans* LET-23/EGFR receptor [21]. These Muv strains are useful for anti-cancer drug screening [21]. In this study using our Muv model, we screened 30 Rabs using RNA interference to find the major regulators of vesicle trafficking supporting Muv formation. Herein, we demonstrate that Rab8 and Rabin8 play a role in Muv formation by maintaining FGFR signaling through fibroblast growth factor (FGF) secretion. We also show that FGFR-TKIs exhibit anti-tumor effects on the EGFR-TKI-resistant, EGFR T790M-L858R-expressing lung cancer cells and nematode model.

## 2. Results

### 2.1. Rab8 Loss-of-Function Mutant Decreases Muv Formation Induced by Hyperactivated EGFR

**Figure 1:**
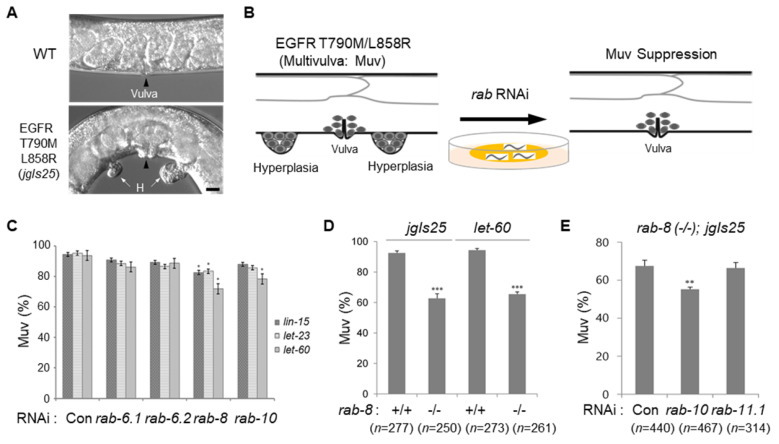
Effects of *rab* RNAi on Muv suppression in *C. elegans* strains with hyper EGFR activity. (**A**) The epidermal growth factor receptor (EGFR) T790M-L858R expressing strain (*jgIs25*) exhibits a tumor-like cell mass (H = hyperplasia indicated by white arrows) in addition to the normal vulva (black arrowhead). It is called multivulva (Muv) because hyperplasia arises from vulval precursor cells (VPCs). Scale bar = 10 μm. (**B**) Screening procedure using 30 *rab* gene knockdown by feeding RNAi to the *jgIs25* strain. (**C**) *C. elegans* Muv mutant strains (*lin-15*, *let-23*, *let-60*) were fed with RNAi for indicated *rab*s and worms with multivulva were counted under the dissecting microscope. More than 200 adult worms were counted in each group. (**D**) Muv strains, *jgIs25* and *let-60,* were cross with *rab-8* (-/-) mutant and worms with multivulva were counted. (**E**) The *rab-8 (-/-); jgIs25* strain fed with RNAi for indicated *rab*s and worms with multivulva were counted. Muv (%) = Percentage of individuals showing multivulva. Error bar = SEM. WT = wild type and Con = control. (*n* = number of worms counted). (* *p* < 0.05, ** *p* < 0.01, *** *p* < 0.001 versus Con). These experiments were performed three times with comparable results.

EGFR mutations such as L858R or T790M-L858R (double mutation) are found in lung cancer patients and are known to be sensitive or resistant to EGFR-TKIs, respectively [1,2,3]. Previously, we established the *jgIs25* transgenic strain, which expresses a LET-23/EGFR gene substituted with the human EGFR T790M-L858R [21]. The *jgIs25* strain shows a multivulva (Muv) phenotype similar to other Muv mutants with multiple tumor-like masses (hyperplasia) in the ventral region (Figure 1A). Vesicle trafficking is important for tumorigenesis induced by growth factor receptor signals such as the EGFR, and Rab proteins are a key regulator of vesicle trafficking [14,22]. For this reason, we performed RNAi screening of 30 *rab* genes to find the positive regulators of vesicle trafficking involved in the Muv formation induced by the mutant EGFR, as described (Figure 1B). We observed that RNAi for several *rab* genes were effective in Muv suppression compared to the control RNAi using the *jgIs25* strain (Figure S1A). To further evaluate these candidates for Muv suppression, we also included several Muv strains involved in EGFR signaling, such as *lin-15* (EGF expression), *let-23* (EGFR), and *let-60* (Ras). The recessive *lin-15* mutant expresses EGF ectopically and displays a strong Muv phenotype [23]. *let-23 (sa62)* and *let-60 (n1046)* are dominant *gf* mutants of EGFR and Ras respectively [20,24]. Interestingly, among these *rab* genes, *rab-8* RNAi resulted in better suppression of Muv formation, although RNAi for *rab-10* showed suppressive effects (Figure 1C).

We further tested whether *rab-8* is important for Muv formation in the activated EGFR or Ras mutants (*jgIs25* and *let-60*). When *rab-8* was deleted, Muv formation was dramatically suppressed in both strains (Figure 1D), indicating that RAB-8 is important for Muv formation when the EGFR pathway is hyperactivated. The *rab-8 (tm2526)* mutant is expected to produce a RAB-8 protein consisting of 62 amino acids, which is estimated as a result of early stop by a 241 bps deletion. Because RNAi for *rab-10* also showed Muv-suppressive effects (Figure 1C) and *rab-10* is known to have redundancy with *rab-8* during embryogenesis and larval development [25], there could be functional redundancy between Rab proteins during vulval development. To test this possibility, *rab-8(-/-); jgIs25* strain was treated with RNAi for *rab-10* and *rab-11*. The Muv ratios of *jgIs25* further decreased by *rab-10* RNAi, but not by *rab-11.1* RNAi in the *rab-8* mutant background (Figure 1E), suggesting a partial redundancy of RAB-8 and RAB-10 during Muv formation.

### 2.2. Rab8 Is Expressed at Vulval Precursor Cells (VPCs) during Vulval Development

**Figure 2:**
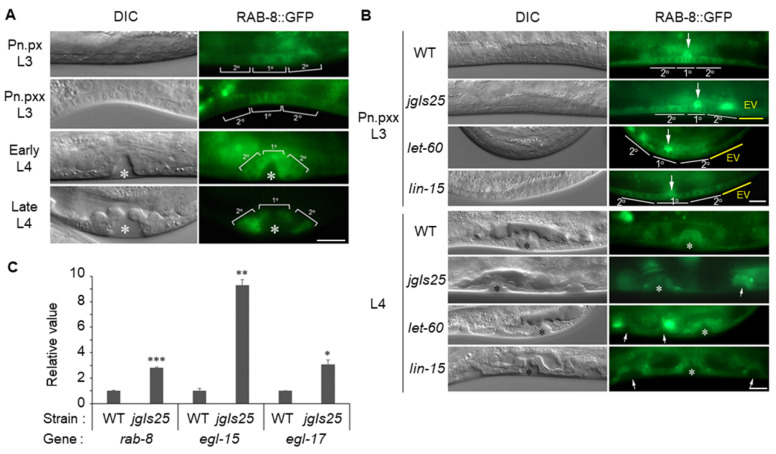
RAB-8 expression in vulval precursor cells (VPCs) during vulval development. (**A**) Transgenic worms expressing RAB-8::GFP by the *rab-8* promoter were observed under the differential interference contrast (DIC) microscope (left panel) and the high resolution fluorescence microscope (right panel). Brackets indicate VPC daughter cells (1^o^ and 2^o^ cells) of L3 (Pn.px and Pn.pxx stages) and L4 (early and late stages) larvae. Asterisks indicate vulval invagination at the L4 larva. DIC = Differential interference contrast image. Scale bar = 10 μm. (**B**) RAB-8::GFP was expressed in WT and Muv strains (*jgIs25*, *let-60gf* and *lin-15*) and DIC images (left panel) and fluorescence images (right panel) were captured at the L3 (Pn.pxx stage, upper panel) and L4 (bottom panel) larvae. White lines indicate VPC daughter cells (1° and 2° cells) that would form a normal vulva and yellow lines indicate extra VPC daughter cells (EV) that could form Muv. Large arrows indicate anchor cells producing LIN-3/EGF. Asterisks indicate normal vulval invagination at the L4 larva, and small arrows indicate extra invagination. Scale bar = 10 μm. (**C**) Total RNAs were extracted from mixed-stage worms of each strain and mRNA levels of *rab-8*, *egl-15*, and *egl-17* genes were measured by qRT-PCR and normalized with actin (*act-1*) expression. Relative expression values compared with WT were shown. Error bar = SD. (* *p* < 0.05, ** *p* < 0.01, and *** *p* < 0.001 versus WT). These experiments were performed three times with comparable results.

Because RAB-8 is important for Muv formation, next we examined *rab-8* expression in vulval precursor cells (VPCs) during vulval development by expression of GFP-conjugated RAB-8 (*rab-8p*::RAB-8::GFP) in the wild-type *C. elegans*. RAB-8::GFP was expressed in the nerves, body wall muscles, intestines, distal tip cells and excretory canal cells (Figure S2), which has been partially described before [26,27]. Interestingly, the RAB-8::GFP proteins were observed at VPCs from the L3 to L4 stages. RAB-8::GFP expression was stronger in 1° cells than in 2° cells in L3 larvae such as the Pn.px and Pn.pxx stages (Figure 2A). The expression pattern of RAB-8::GFP in 1° cells in L3 larvae is similar to that of EGL-17, a nematode fibroblast growth factor (FGF) homolog, which is expressed highly in 1° cells in L3 and in 2° cells in L4 [28,29].

Next, we examined whether RAB-8 expression was affected by the elevated EGFR/Ras/MAPK activity using Muv strains, *jgIs25*, *let-60gf*, and *lin-15*. There was little difference in the RAB-8 expression patten in the VPCs between the wild type and Muv mutants (Figure 2B). Interestingly, RAB-8 was also expressed in the ectopic VPC daughter cells as indicated with a yellow line in the upper panel and small arrows in the bottom panel (Figure 2B). Similarly, EGL-17/FGF is known to be expressed in the ectopic VPCs in Muv mutants [29]. Therefore, we investigated the expression levels of *rab-8*, *egl-15* and *egl-17* in the *jgIs25* Muv strain using qRT-PCR. EGL-17 and EGL-15 are *C. elegans* orthologs of FGF and FGFR, respectively. The mRNA levels of these genes increased significantly in *jgIs25* compared to the wild type (Figure 2C). These results indicate that the hyperactivated EGFR signal of *jgIs25* increased the FGFR signal through overall upregulation of *rab-8*, *egl-15*, and *egl-17*.

### 2.3. The C. elegans Rabin8 Functions in Muv Formation

**Figure 3:**
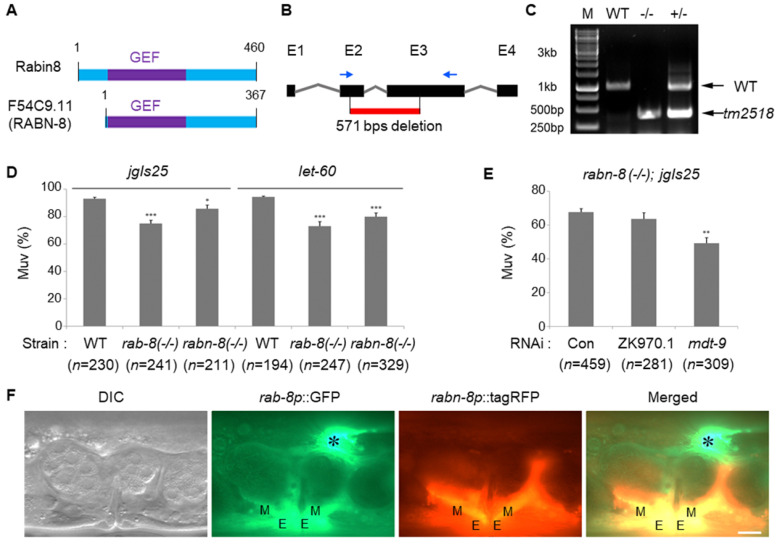
*C. elegans* Rabin8 ortholog RABN-8 functions in Muv formation. (**A**) Primary structure of human Rabin8 and *C. elegans* RABN-8 (F54C9.11) proteins. The conserved GEF domain and polypeptide length of each protein are indicated. (**B**) Genomic structure of *rabn-8.* The deletion site (red line) of the *rabn-8* (*tm2518*) mutant and 4 exons (E1 to E4) are shown. Blue arrows indicate primer sites used for genotyping. (**C**) Wild type (WT), *tm2518* homozygote (-/-), and heterozygote (+/-) were genotyped by genomic DNA PCR using the primers indicated in B (blue arrows). M = size marker. (**D**) Muv strains (*jgIs25* and *let-60*) were crossed with *rab-8* or *rabn-8* mutants, and worms with Muv were counted. Error bar = SEM. (* *p* < 0.05 and *** *p* < 0.001 versus WT). (**E**) The *rabn-8*; *jgIs25* strain was fed with RNAi of the MSS4 homologue ZK970.1 or *mdt-9* and worms with Muv were counted. Error bar = SEM. (** *p* < 0.01 versus Con). These experiments were performed three times with comparable results. (**F**) Transgenic worms containing *rab-8p*::GFP and *rabn-8p*::tagRFP expression plasmids were observed. Asterisk = distal tip cell. M = vulval muscle. E = vulval epithelia. Scale bar = 10 μm.

Rab activity is regulated either negatively or positively by GTPase-activating proteins (GAPs) or guanine nucleotide exchange factors (GEFs), respectively. Although Rabin8 has been reported to have Rab8-specific GEF activity in humans, there is no report on the regulation of RAB-8 in *C. elegans* [30]. Therefore, we examined the *C. elegans* Rabin8 candidate gene and found that the *C. elegans* Rabin8 ortholog (RABN-8) is predicted to be encoded from the *F54C9.11* gene. RABN-8 has a GEF domain with a short N-terminal region compared to human Rabin8 (Figure 3A). The *tm2518* mutation contains a 571 bps deletion in the *F54C9.11* locus, which is an *rabn-8* loss-of-function *(lf)* mutation (Figure 3B). The *rabn-8 (tm2518)* mutation is expected to produce a protein with 66 amino acids as a result of early stop. After crossing with wild-type worms four times, the outcrossed *rabn-8 (tm2518)* mutant was analyzed by genomic DNA PCR using the indicated primers (blue arrows) (Figure 3B,C) and used for further experiments to test whether *rabn-8* affects Muv development.

The Muv ratio of *jgIs25* and *let-60* was reduced in the *rabn-8* mutant compared to *jgIs25* or *let-60* alone (Figure 3D). As the decrease of Muv formation by the *rabn-8* mutation was less than that by the *rab-8* mutation, we reasoned that there might be other positive regulators of RAB-8 activity in *C. elegans*. To test this possibility, *rabn-8; jgIs25* was treated with the RNAi of MSS4 homologous genes, which are known to stabilize several Rab proteins in mammals [31]. *C. elegans* has two MSS4 homologs, namely, ZK970.8 and *mdt-9*, and we found that *mdt-9* knockdown by RNAi additionally reduced the Muv formation of *rabn-8* (-/-)*; jgIs25* (Figure 3E). These results indicate that *C. elegans* Rab8 is also regulated by a variety of proteins, similar to the mammalian Rab8 protein. To investigate the expression patterns of *rabn-8* and *rab-8*, tagRFP and GFP reporters were expressed by the *rabn-8* and *rab-8* promoters, respectively. At the overall individual level, *rab-8p*::GFP was expressed in more cells and tissues than r*abn-8p*::tagRFP (Figure S3). Expectedly, expressions of *rabn-8p*::tagRFP and *rab-8p*::GFP overlapped in vulval cells, including muscle and epithelial cells, but not in the distal tip cells where *rab-8p*::GFP was highly expressed (Figure 3F).

### 2.4. RAB-8 and RABN-8 Formed a Complex and Colocalized

**Figure 4:**
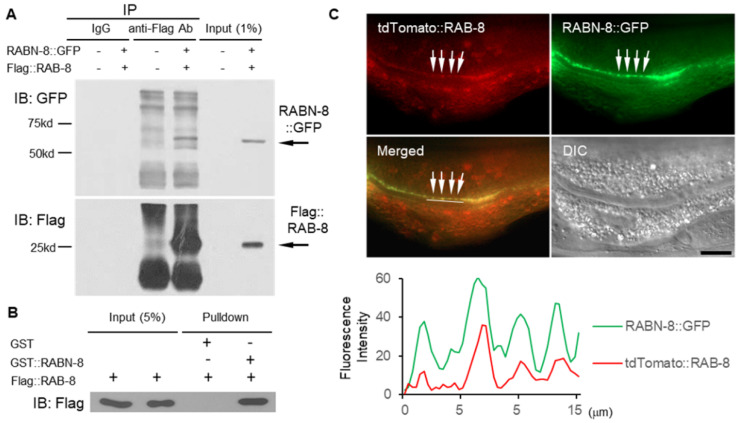
Direct interaction and colocalization of RAB-8 and RABN-8. (**A**) HEK293 cells were transiently transfected with the expression plasmids of GFP::RABN-8 and Flag::RAB-8. Cell lysates were immunoprecipitated (IP) with anti-Flag antibody and immunocomplexes were processed for immunoblot (IB) analysis using anti-GFP (upper panel) or anti-Flag (bottom panel) antibodies, respectively. Mouse IgG was used as a negative control. The minus sign (-) denotes vector control for each RABN-8::GFP and Flag::RAB-8 transfected cell lysate. (**B**) In vitro GST pulldown experiment of RAB-8 and RABN-8. Immunoblotting was performed with the anti-Flag antibody to detect Flag::RAB-8 proteins after pulling down to GST or GST::RABN-8. (**C**) Transgenic worms expressing tdTomato::RAB-8 and RABN-8::GFP were observed, and RABN-8::GFP and tdTomato::RAB-8 proteins located near the apical membrane of the intestine are indicated by arrows. The fluorescence fusion protein was expressed in the intestine by the *vha-6* promoter. The intensity of fluorescence measured along the white line indicated in the merged image is graphically displayed (bottom panel). Scale bar = 10 μm.

To investigate a possible interaction between RAB-8 and RABN-8, immunoprecipitation (IP) was performed using Flag::RAB-8 and GFP::RABN-8, epitope-tagged nematode proteins expressed in HEK293 cells. Intriguingly, GFP::RABN-8 was found in the Flag::RAB-8 immunocomplex, indicating their complex formation in vivo (Figure 4A). To further determine whether RAB-8 forms a complex with RABN-8 in vitro, GST pulldown analysis was carried out via Flag::RAB-8 and GST::RABN-8 expression in HEK293 and *Escherichia coli*, respectively. When the purified GST or GST::RABN-8 were incubated with cell lysates from HEK293 cells expressing Flag::RAB-8, Flag::RAB-8 was detected in GST::RABN-8, but not in the GST control, suggesting a complex formation of RAB-8 with RABN-8 (Figure 4B).

We also investigated the localization of RAB-8 and RABN-8 using the *vha-6* promoter to express these proteins in intestinal cells, which are large cells whose cell biology and trafficking are well established [32]. Both tdTomato::RAB-8 and GFP::RABN-8 proteins were located at the same position near the apical membrane of the intestinal cells (Figure 4C). According to the fluorescence image data in the top panel, the graphical representation of the intensity measurements of RABN-8::GFP and tdTomato::RAB-8mostly overlapped each other (bottom panel), indicating that they are colocalized.

### 2.5. RABN-8 Is Required for FGF Secretion in VPCs

**Figure 5:**
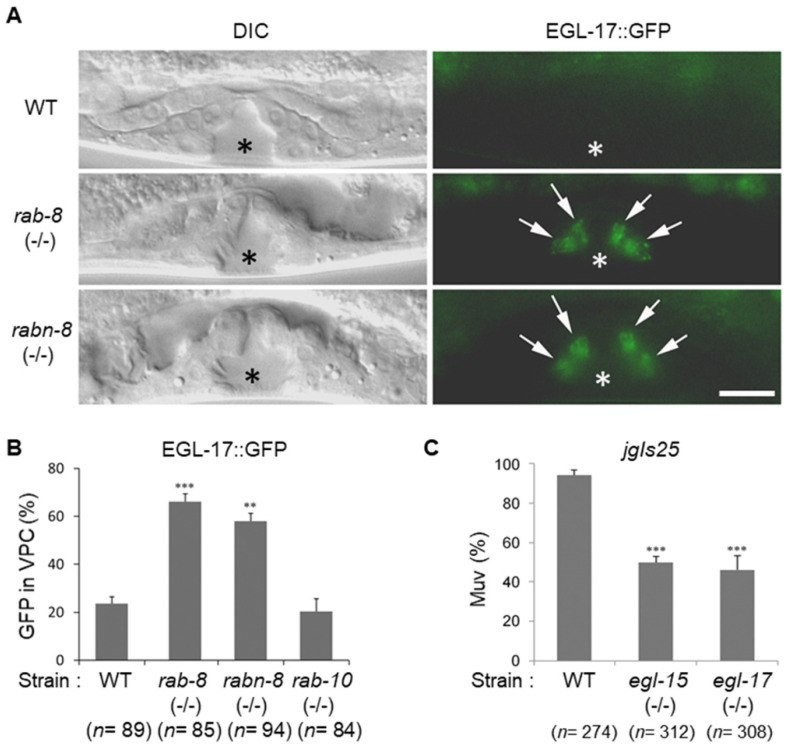
Function of RAB-8 and RABN-8 in EGL-17::GFP secretion. (**A**) EGL-17::GFP expression in the vulval region of either WT, *rab-8* mutant, or *rabn-8* mutant worms were observed. Arrows indicate EGL-17::GFP protein expressed in VPC daughter cells. The green signal in the intestine is autofluorescence of gut granules. Asterisks indicate vulval invagination. Scale bar = 10 μm. (**B**) EGL-17::GFP-positive worms in each group were counted. Error bar = SEM. (**C**) The *jgIs25* strain was crossed with the FGFR or FGF mutants (*egl-15* or *egl-17* respectively), and worms with Muv were counted. Error bar = SEM. (** *p* < 0.01, *** *p* < 0.001 versus WT). These experiments were performed three times with comparable results.

Because RAB-8 is known to be important for FGF secretion, RABN-8 is expected to be required for FGF secretion if RABN-8 functions as a RAB-8 GEF in VPCs [33]. Therefore, EGL-17::GFP was used to investigate whether the secretion of EGL-17, a *C. elegans* FGF, was altered in the VPCs of *rab-8* and *rabn-8* mutant animals. The fluorescent intensity of EGL-17::GFP was weak in the VPCs of the wild-type animals because it was secreted from the cells, as previously reported [33]. In contrast, the intensity of EGL-17::GFP was much higher in the VPCs of the *rab-8* and *rabn-8* mutants than in the wild type (Figure 5A). When counting the number of L4 larvae that exhibited EGL-17::GFP accumulation in VPCs, more than 60% of *rab-8* and *rabn-8* mutants were GFP-positive, whereas 20% of wild-type animals were GFP-positive (Figure 5B). Although RAB-10 functioned redundantly with RAB-8 in Muv formation as shown previously (Figure 1D), the number of EGL-17::GFP-positive larvae in the *rab-10* mutant was similar to that of the wild type (Figure 5B).

RAB-8 and RABN-8 function in EGL-17/FGF secretion from the VPCs (Figure 5A,B) and the FGF signaling pathway plays an important role in vulval development [18,33,34]. We reasoned that the requirement of RAB-8 in Muv formation might be due to its role in regulating EGL-17/FGF signaling. Therefore, we examined whether FGF and the FGFR are necessary for Muv formation in the hyperactivated EGFR-expressing *jgIs25* strain. Very interestingly, the proportion of Muv-bearing animals was significantly reduced in the *egl-15* and *egl-17* mutants, which are loss-of-function mutants for the FGFR and FGF, respectively (Figure 5C). Muv formation in the *jgIs25* strain was more effectively decreased in the *egl-15* and *egl-17* mutants than in the *rab-8* mutant (Figure 1C). These data suggest that FGFR signaling, which is partially regulated by RAB-8 and RABN-8 through FGF secretion, is critical for Muv formation induced by activated EGFR mutations.

Secreted FGF acts on sex myoblasts (SMs) through the FGFR and functions in the SM migration and morphogenesis of vulval muscles [35]. SMs migrate from the posterior body to the vulva to form vulval muscles that open the vulva for egg-laying [28]. We investigated the migration of SMs in the *rab-8* mutant using an *egl-15p*::GFP transgene, which expresses highly in SMs, as previously reported [36]. There was little difference in the *egl-15p*::GFP expression pattern and SM migration between the wild type, *rab-8* and *jgIs25* strains in both L4 and adult stages (Figure S4).

### 2.6. FGFR Inhibitors Effectively Reduced Muv Formation in the EGFR-TKI-Resistant Model

**Figure 6:**
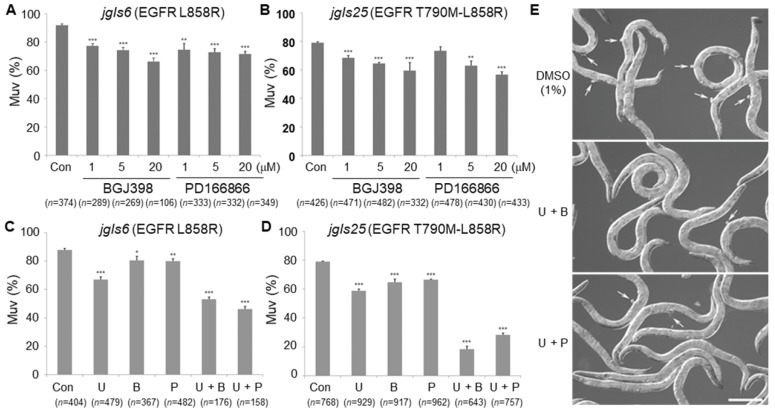
Effects of FGFR and MEK inhibitors on Muv formation. (**A**,**B**) Muv worms (*jgIs6 and jgIs25*) were treated with indicated concentrations of FGFR inhibitors, BGJ398 and PD166866, and then worms with Muv were counted. The *jgIs6* and *jgIs25* strains express the EGFR-TKI-sensitive EGFR L858R and the EGFR-TKI-resistant EGFR T790M-L858R, respectively. (**C**,**D**) Muv worms (*jgIs6 and jgIs25*) were treated either with 1 μM MEK inhibitor U0126 (U), 5 μM BGJ398 (B), 5 μM PD166866 (P), 1 μM U + 5 μM B, or 1 μM U + 5 μM P, and worms with Muv were counted. (**E**) DIC images of the *jgIs25* strain treated with indicated drugs. Arrows indicate Muv. Scale bar = 200 μm. U = U0126. B = BGJ398. P = PD166866. Error bar = SEM. (* *p* < 0.05, ** *p* < 0.01, and *** *p* < 0.001 versus Con). These experiments were performed three times with comparable results.

Next, to investigate whether FGFR inhibition reduces Muv formation, the *C. elegans* Muv model expressing the mutant EGFRs was treated with two FGFR inhibitors. We employed *jgIs6,* another strain expressing the EGFR L858R mutant, which is sensitive to EGFR-TKIs [21]. BGJ398 (Infigratinib) is a pan FGFR-TKI and PD166866 is a selective FGFR1-TKI [37,38,39]. The FGFR inhibitors (FGFR-TKIs) reduced the Muv ratio of *jgIs6* and *jgIs25* in a dose-dependent manner, although BGJ398 appeared to be more effective than PD166866 (Figure 6A,B). These results further indicate that FGF signaling contributes to ectopic cell proliferation when the EGFR pathway is hyperactive.

Previously, we showed that the MEK inhibitor U0126 inhibited Muv formation in the EGFR-TKI-resistant *jgIs25* strain [21]. We examined whether cotreatment of FGFR inhibitors and U0126 synergistically reduces Muv formation. The combination of these inhibitors effectively decreased Muv formation in *jgIs6* and *jgIs25* and these inhibitory effects were augmented in the EGFR-TKI-resistant *jgIs25* than in the EGFR-TKI-sensitive *jgIs6* (Figure 6C–E). Single treatment of 1 µM of U0126 reduced the Muv ratio by 20% in *jgIs6* and *jgIs25,* but the combination treatment of 1 µM of U0126 and 5 µM of BGJ398 decreased the ratio by 40% and 60% in *jgIs6* or *jgIs25*, respectively (Figure 6C,D). These results indicate that the inhibition of both FGFR and MEK is more effective for suppressing Muv formation in the EGFR-TKI-resistant model.

### 2.7. FGFR-TKI Inhibits the Growth of EGFR-TKI-Resistant Lung Cancer Cells

**Figure 7:**
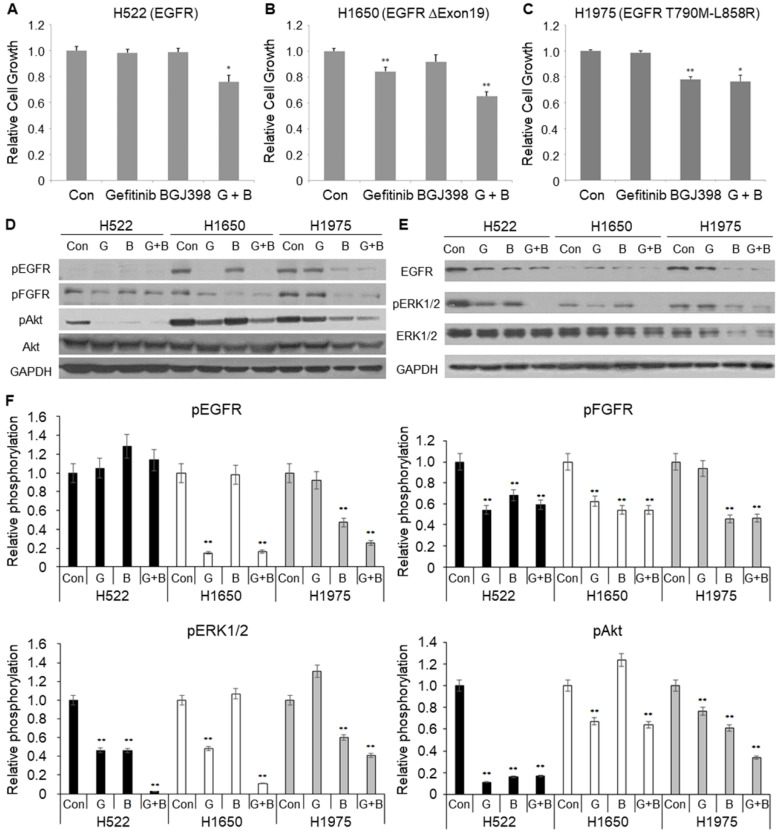
Effects of EGFR-TKI and FGFR-TKI on growth of lung cancer cell lines with different EGFR mutations. (**A**–**C**) Cells treated with 5 μM gefitinib (G), 10 μM BGJ398 (**B**) or two together (G + B) for 24 hours and cell growth was assessed by MTS assay. Error bar = SEM. (**D**,**E**) Cells treated as in (**A**–**C**) were lysed and each 20 μg of total protein was analyzed by immunoblotting using the indicated antibodies. GAPDH was used as a loading control. (**F**) Levels of pEGFR, pFGFR, pERK1/2, and pAkt were analyzed by densitometry and normalized with GAPDH. Fold changes relative to control were presented. Relative phosphorylation indicates relative fold of phosphorylation level of each protein. Error bar = SD. (* *p* < 0.05 and ** *p* < 0.01 versus Con). These experiments were performed three times with comparable results.

The EGFR T790M-L858R mutant was initially found in human lung cancer patients with EGFR-TKI resistance [1,2]. Therefore, we further tested whether the inhibition of both the EGFR and the FGFR together attenuates the growth of human lung cancer cells. We employed three human lung cancer cell lines, namely, H522, H1650, and H1975, which express the wild-type EGFR, 19th exon-deleted EGFR, and EGFR T790M-L858R, respectively [5]. Deletion in the 19th exon of the EGFR is one of the major activation mutations found in lung cancer patients [40]. As expected, gefitinib, an EGFR-TKI, reduced growth of H1650 cells but it was less effective in both H522 and H1975 cells. BGJ398, an FGFR-TKI, inhibited cell growth more effectively in H1975 cells than any other cell line. When cells were co-treated with gefitinib and BGJ398, cell growth inhibition was synergistically increased in both H522 and H1650 cells (Figure 7A–C). However, in H1975 cells expressing EGFR T790M-L858R, the growth inhibition by cotreatment was similar to that of BGJ398 alone (Figure 7C).

Next, we examined changes in the cellular signaling pathways affected by gefitinib and BGJ398. Activities of EGFR and FGFR were assessed by antibodies recognizing their specific tyrosine phosphorylations. Gefitinib decreased pEGFR levels in H1650, but not in H1975 cells (Figure 7D). It is very interesting that BGJ398 decreased pEGFR or total EGFR as well as pFGFR to a greater or lesser extent in the three cell lines (Figure 7D,E and Figure S5). It is intriguing that BGJ398 dramatically decreased pFGFR, pEGFR, and total EGFR by inhibiting the growth of EGFR-TKI-resistant H1975 cells (Figure 7C–E) because these results are consistent with Muv inhibition by BGJ398 in the EGFR-TKI-resistant nematode (Figure 6). Consistent with synergistic effects in H522 and H1650 cells, gefitinib and BGJ398 together dramatically decreased pERK1/2 levels in those cells, but not in H1975 cells which were not sensitive to cotreatment (Figure 7D,E). Although both gefitinib and BGJ398 decreased pAkt levels, cotreatment did not further decrease pAkt in the three cell lines. It is probable that gefitinib and BGJ398 synergistically inhibit cell growth via inhibition of crosstalk between the EGFR and the FGFR, which leads to the inactivation of downstream signaling molecules, such as pERK1/2 and pAkt. All of these data suggest that FGFR-TKIs could be a possible solution to EGFR-TKI-resistant cancers expressing EGFR T790M-L858R. In addition, the combination of EGFR-TKIs and FGFR-TKIs can be expected to be a better anticancer strategy than single TKI treatment of cancers with hyper activities of the EGFR and the FGFR.

## 3. Discussion

We investigated Rab8 and Rabin8 function using the *C. elegans* tumor model to understand the role of vesicle trafficking during multivulva development induced by EGFR T790M-L858R. Our results suggest that several Rab proteins act redundantly under the EGFR downstream. Some Rab proteins are known to affect EGFR trafficking directly, such as Rab5, 7 and 11 [15]. However, RAB-8 functions in the EGFR downstream rather than EGFR trafficking directly, because LET-23/EGFR::GFP localization did not change in VPCs of the *rab-8* mutant (Figure S1B). In addition, *rab-8* mRNA expression was increased by hyperactivation of the EGFR (Figure 2C), and the *rab-8* (-/-) and *rabn-8 (-/-*) mutants suppressed the Muv phenotype of the *let-60/Ras gf* mutant (Figure 1D and Figure 3D) downstream of the EGFR in vulval development.

We found that the FGFR signaling pathway mediates the oncogenic EGFR signal from the result that Rab8 and Rabin8 are required for the secretion of FGF in nematode vulval development. In particular, the hyperactivated EGFR enhanced the mRNA expression of *rab-8*, *egl-15* and *egl-17* together (Figure 2C). These results suggest that hyperactivation of the EGFR increases FGF signals and secretion simultaneously. However, even the *rab-8 lf* mutant partially blocked FGF secretion, and the Muv suppression effects were significantly lower than those of the *egl-15* and *egl-17* mutants. Therefore, RAB-8 is partially involved in the regulation of FGF secretion. Because SMs migrate normally in the *rab-8* mutant (Figure S4A), the amount of FGF required for SM migration appears to be secreted. Several Rab proteins, such as RAB-5 and RAB-7 have been reported to be involved in FGF secretion, so the redundancy between Rab proteins seems to work even when FGF is secreted [33]. However, RAB-10, which is the most functionally close to RAB-8, is not involved in FGF secretion (Figure 5B). In addition, because there is non-traditional FGF secretion that does not occur through traditional vesicle trafficking, FGF secretion through other pathways cannot be excluded [41]. For this reason, the *rab-8* mutation seems to have a less of inhibitory effect on Muv than the FGF or FGFR mutations.

Both the EGFR and FGFR signal transduction pathways are important in cancer, and the FGFR pathway is activated by EGFR-TKI treatment [11]. Therefore, the activation of the FGFR is likely to be another cause of resistance to EGFR-TKIs, and the combination of FGFR-TKIs and EGFR-TKIs is effective in glioblastoma and non-small cell lung cancer, as previously reported [42,43]. Since the FGFR mediates EGFR signaling, the efficacy of FGFR-TKIs in cancer treatment was investigated using *C. elegans* tumor models and lung cancer cell lines. The effect of U0126 was dramatic in the nematode tumor model, but not in the lung cancer cell lines. It is assumed that the nematode tumor model does not fully reflect the characteristics of a cancer cell line. In contrast, in lung cancer cells with a KRAS-mutation during the epithelial-to-mesenchymal transition, MEK inhibition activates the FGFR, so it has been reported that cotreatment of FGFR-TKIs and MEK inhibitors effectively inhibits cancer cell survival and tumor growth [44]. Similarly, cotreatment with MEK inhibitors and FGFR-TKIs showed a synergistic effect in the EGFR-TKI-resistant nematode model (Figure 6D).

In EGFR-TKI-resistant cancers, EGFR-TKI and FGFR-TKI combination treatment is no more beneficial for inhibiting cell growth than FGFR-TKIs alone (Figure 7C). These results can provide a basis for using EGFR-TKIs and FGFR-TKIs alone or in combination depending on the type of cancer. Contrary to this result, it has been reported that double inhibition of the EGFR and the FGFR in non-small cell lung cancer inhibit the survival and expansion of drug-resistant cells with EGFR mutations over a long period of time [42]. In addition, as the FGFR is a major target pathway in the tumor microenvironment, FGFR-TKIs are a promising anticancer drug with many benefits [45,46]. Interestingly, in this study, BGJ398 alone reduced both pEGFR and total EGFR in the H1975 cell line expressing EGFR T790M-L858R. In a study using another FGFR-TKI, AZD4547, in the H1975 cell line, AZD4547 lowered pEGFR but did not reduce the amount of total EGFR [47]. These studies, with some differences, suggest that FGFR-TKIs are effective in overcoming EGFR-TKI-resistant cancer cells.

**Figure 8:**
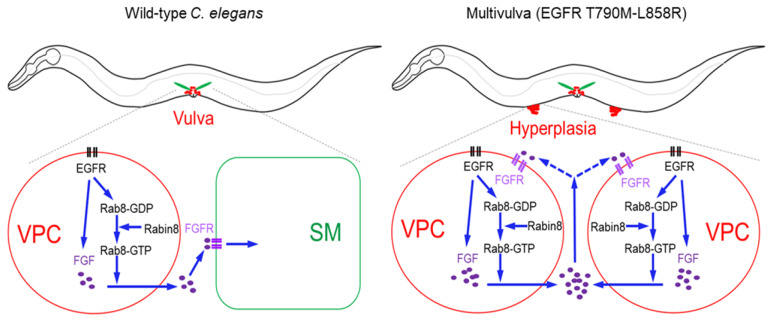
Proposed model of Rab8 and Rabin8 for Muv formation in *C. elegans* expressing a hyperactive human EGFR mutant. In the wild-type worm, EGFR signaling in VPCs induces FGF expression and its secretion via Rab8 and Rabin8. Secreted FGF acts on sex myoblast (SM) cells expressing FGFR, and thus induces migration and differentiation of SM to form vulval muscles (left panel). In the multivulva worms expressing a hyperactive EGFR mutant, excessive EGFR signaling in VPCs results in increased FGF production and secretion, leading to Muv formation in addition to the normal vulva. Autocrine or paracrine of FGF signaling may act among daughter cells of extra vulval precursor cells (right panel).

EGL-15/FGFR is expressed in vulval muscle cells rather than in vulval epithelial cells [48]. It appears that the regulation of FGF secretion by RAB-8 does not seem to be a problem for the normal development of the vulva in the *rab-8* mutant in the context of wild-type EGFR signaling. In contrast, Muv formation induced by hyperactive EGFR signaling seems to require a higher level of FGF secretion. The SM migration and morphogenesis were normal in the *rab-8* mutant (Figure S4A). FGFR signaling functions in peripheral tissues such as vulval muscles and may affect the cell proliferation of VPCs in a non-autonomous manner. Therefore, Muv inhibition in *jgIs6* or *jgIs25* by the FGFR inhibitor BGJ398 may result from the decrease of FGFR activity in peripheral cells expressing EGL-15/FGFR. However, since the hyperplasia site formed in the Muv model is far from SMs, it will not be able to receive support from the vulval muscle in contrast to the normal vulva. Based on these results, models of Rab8, Rabin8, and FGFR signaling during nematode Muv development were presented (Figure 8). In the wild-type worm, the FGF produced in VPCs is transported by RAB-8. Then, FGF transmits a signal to SM cells expressing the FGFR to achieve normal migration and differentiation of SMs to form vulval muscles. In the Muv strain expressing EGFR T790M-L858R, hyperactivated EGFR signals increase RAB-8/Rab8 and EGL-17/FGF expression. Since the hyperplasia site is far from SMs, there is a possibility that autocrine or paracrine may act between ectopic VPC daughter cells, despite the failure to observe EGL-15 expression at the hyperplasia site (Figure S4).

We suggest that the function of Rab8 and its regulator Rabin8 mediate the oncogenic signaling of the EGFR through FGF secretion in the *C. elegans* Muv model expressing the hyperactivated and EGFR-TKI-resistant EGFR T790M-L858R. This study suggests that the combination of EGFR-TKIs and FGFR-TKIs is effective for lung cancer cells, thus providing conceptual support for the expansion of the existing EGFR-TKIs. Above all, FGFR-TKIs show the potential for application in the treatment of EGFR-TKI-resistant cancer cells.

## 4. Materials and Methods

### 4.1. C. elegans Culture and Strains

*C. elegans* was cultured on nematode growth media (NGM) as described previously [49]. Mutant strains used in this study are *egl-15(n484)*, *egl-17(e1313)*, *let-23(sa62)*, *let-60(n1046)*, *lin-15(n765)*, *rab-8(tm2526)*, *rab-10(ok1494)*, and *rabn-8(tm2518)*. These strains were provided by *Caenorhabditis* Genetic Center (CGC, Minesota University, Minneapolis, MN, USA) and National Bioresource Project (NBRP, Tokyo, Japan). The integration lines *jgIs6* (*let-23p*::LET-23::EGFR L858R; pRF4 (*rol-6*)) and *jgIs25* (*let-23p*::LET-23::EGFR T790M-L858R; pRF4 (*rol-6*); pCFJ90 (*myo-2p*::mCherry)) were used in the previous study [21]. The *jgIs40* (*egl-17p*::EGL-17::GFP; pCFJ90) and *jgIs42* (*rab-8p*::GFP::RAB-8; pCFJ90) strains were generated by UV irradiation.

### 4.2. Bacteria Feeding RNA Interference

Most RNAi clones were from Ahringer library and kindly provided by Dr. J. Lee’s laboratory (Seoul National University). Other *rab* RNAi clones were made by cDNAs from RT-PCR using N2 RNAs and specific primer sets. The amplified PCR fragment was cloned into the L4440 vector using a restriction enzyme site tagged to each primer. The names and sequences of the primers are listed in a supplementary data (Table S1). RNAi clones of ZK970.8 and *mdt-9* were produced in the same manner as *rab* RNAi cloning using cDNA of each gene. These RNAi plasmids were transformed into HT115 *E. coli* strain. RNAi plates were prepared using general 60 mm NGM plates where 1 mM IPTG (Duchefa, I1401.0005) and 50 µg/ml ampicillin (Amresco, 3370C469) were added. Most *rab* RNAi started at L4 and counted the Muv of the F1 progeny, but some *rab* RNAi was performed from the L1 larva because their knockdown resulted in embryonic lethality. 

### 4.3. Reporter Gene Construction, Microinjection, Integration and Microscopy

To produce reporter constructs and expression plasmids, PCR was carried out using specific primer pairs and N2 genomic DNA or cDNA as templates. The amplified DNA fragment was cloned into a suitable vector using restriction enzymes. The plasmids, vectors, and primers used in these experiments are shown in a supplementary data (Table S1). Each plasmid concentration for microinjection is 30 to 50 µg/ml, and total mixture concentration was adjusted to 150 µg/ml, including transgenic markers such as 50 µg/ml pRF4, 5 µg/ml pCFJ90, and 50 µg/ml *unc-119* (+). The stable lines were selected at F2 generation and used for further experiments. Integration lines including *jgIs40* and *jgIs42* were generated by UV irradiation. Each integration line was outcrossed with wild-type worms 4 times to exclude extra mutations. For DIC and fluorescence imaging, worms were prepared on 2% agarose slides with M9 buffer and 2 mM levamisole (Sigma, L9756, St. Louis, MO, USA). Photographs of worms were captured using a DFC420C camera (Leica) attached to an Axio Imager A1 microscope (Zeiss).

The intensity of the fluorescent protein was measured using the ImageJ program (https://imagej.net/). The measurements of the marked area were achieved by the plot profile and displayed graphically after background subtraction.

### 4.4. Immunoprecipitation and GST-Pulldown Analysis

For the immunoprecipitation, *rabn-8* cDNA was cloned into pEGFP-C1 (GeneBank Accession #: U55763) and *rab-8* cDNA was cloned into the pCMV-3Tag-1A vector (Agilent Technologies, 240195). GFP::RABN-8 or Flag::RAB-8 expression plasmids were transfected to HEK293 cells together. After washing with ice-cold PBS (10 mM Na_2_HPO_4_ pH 7.4, 145 mM NaCl, and 2.7 mM KCl), cells were lysed with RIPA buffer (25 mM Tris-HCl, pH 7.5, 150 mM NaCl, 1% Nonidet P-40, 0.1% SDS, 1% sodium deoxycholate, 1 mM PMSF, protease inhibitor). Solubilized lysates were incubated with specific antibody or preimmune IgG at a final concentration of 1 μg/ml each for overnight at 4 °C and cell lysates were immunoprecipitated with mouse IgG and GFP (Santa Cruz, CA, USA) antibodies or with mouse IgG and Flag (Santa Cruz, CA, USA) antibodies and incubated overnight at 4 °C. Protein A/G agarose beads were added and incubated for 2 hours and washed three times with IP lysis buffer and immunoprecipitated samples were processed for immunoblotting experiments.

For the GST-pulldown experiment, GST and GST::RABN-8 was expressed in BL21 *E. coli* strain using the pGEX-4T-1 vector (Sigma, GE28-9545-49, St. Louis, MO, USA) and pGEX-4T-1 with *rabn-8* cDNA. We cultured BL21 at 20 °C and induced by adding 0.2 mM IPTG for 4 hours to achieve soluble proteins. Bacteria were harvested by centrifuge at 4 °C, and then resuspended with sonication buffer (50 mM Tris-HCl, 200 mM NaCl, 1 mM DTT, and 1 mM PMSF) with protease inhibitors (Roche, 11836153001). The supernatant fraction was added to glutathione agarose beads (Sigma, G-4510, St. Louis, MO, USA) after centrifugation of sonicated bacterial lysates. After rocking in the cold room for overnight, the beads were washed 4 times with sonication buffer. We ketp them at 4 °C until Flag::RAB-8 proteins were prepared. Flag::RAB-8 expressing plasmid was transfected to HEK293 cells, and cell lysates was added to GST-bound or GST::RABN-8-bound glutathione beads with rocking in the cold room for overnight. The next morning, we washed 4 times with sonication buffer and performed Western blot analysis using anti-Flag antibody. The plasmids, vectors, and primers used in these experiments are shown in a supplementary data (Table S1). The original image of GST pulldown result is shown in a supplementary figure (Figure S6).

### 4.5. Drug Treatment and Statistics in C. elegans

Treating compounds to *C. elegans* using 96-well plates was followed as the previous method for drug treatment [21]. The synchronized L1 larvae on the normal NGM plate with cholesterol were harvested using M9 buffer and washed 3 times, then transferred to each well of the 96-well plate. The final culture volume was adjusted to 100 µl and worm numbers were between 50–100 per well. OP50 *E. coli* was cultured at 37 °C, harvested, washed 2 times with distilled water, and kept at 4 °C. L1 larvae cultured on the 96-well plate were incubated for 3 days at 22 °C and transferred to NGM plates. Counting Muv was performed using a dissecting microscope after 24 hours of recovery. BGJ398 (S2183, Selleck Chemicals LLC, Houston, TX, USA) and PD166866 (S8493, Selleck Chemicals LLC), and U0126 (036M4607V, Sigma, St. Louis, MO, USA) were used. These experiments were repeated more than 3 times and *p*-value to the control (* *p* < 0.05, ** *p* < 0.01, and *** *p* < 0.001) was calculated by *t*-test.

### 4.6. Cell Culture, MTS Assay and Immunoblotting

Cancer cells were grown in RPMI-1640 media (SH30027.01, Hyclone) containing 10% fetal bovine serum (FBS) and 1x antibiotics at 37 °C using an incubator supplied with 5% CO2 concentration. The drugs were diluted in RPMI-1640 media containing 1% FBS but without antibiotics and treated with 100 ml volumes in cancer cells. Each cancer cell line was treated with 5 μM of gefitinib (S1025, Selleck Chemicals LLC, Houston, TX, USA) and 10 μM of BGJ398 alone or in combination.

In vitro 3-(4,5-dimethylthiazol-2-yl)-5-(3-carboxymethoxyphenol)-2-(4-sulfophenyl)-2H-tetrazolium (MTS) assay was performed to evaluate cell viability using a commercially available kit (Promega, ADG3581). Initially, 4000 to 5000 cells were cultured in each well of 96-well tissue culture plates (NCI-H1975 and NCI-H522 = 4000 cells/well, NCI-H1650 = 5000 cells/well), and drugs were added the following day. After 24 hours of drug treatment, 20 μl of MTS solution (CellTiter 96 Aqueous One Solution Cell Proliferation Assay; Promega, Madison, WI, USA) was added. The cells were incubated for an additional 4 hours (37 °C, 5% CO2), and the absorbance was measured at 490 nm using an ELISA plate reader (Tunable VERSA maxmicroplate reader, Molecular Devices, San Jose, CA, USA).4.7. Western blot analyses and reagents.

NCI-H522, NCI-H1650, and NCI-H1975 lung adenocarcinoma cells were treated without or with the EGFR inhibitor, 5 μM of Gefitinib and FGFR inhibitor, 10 μM BGJ 398 for 24 h, and cell lysates from NCI-H522, NCI-H1650, and NCI-H1975 cells were analyzed by immunoblotting using indicated antibodies. After washing with ice-cold PBS (10 mM Na2HPO4 pH 7.4, 145 mM NaCl, and 2.7 mM KCl), cells were lysed with 2× sodium dodecyl sulfate (SDS)-PAGE sample buffer (20 mM Tris pH 8.0, 2% SDS, 2 mM DTT, 1 mM Na3VO4, 2 mM EDTA, and 20% glycerol) and boiled for 5 min. The protein concentration of each sample was determined by using the micro-bicinchoninic acid protein assay reagent as described by the manufacturer (Thermo Fisher Scientific, Waltham, MA, USA). Total cellular protein (30 μg/lane) was separated by 10% SDS-PAGE and transferred to polyvinylidene difluoride (PVDF) membranes. Membranes were blocked overnight at 4 °C in TBST (20 mM Tris pH 8.0, 150 mM NaCl, and 0.05% Tween 20) containing 5% non-fat milk. Membranes were then incubated overnight at 4 °C with primary antibody, washed three times with TBST, incubated with horseradish peroxidase (HRP)-conjugated goat anti-rabbit IgG secondary antibody for 1 h at room temperature, and washed three times with TBST. Proteins were visualized using an enhanced chemiluminescence reagent (Millipore, Burlington, MA, USA). Polyclonal antibodies against phospho-EGFR, phospho-FGFR, phospho-ERK, ERK, phospho-AKT, AKT, LC3, GAPDH, and vinculin were purchased from Cell Signaling Technology (Beverly, MA, USA). The EGFR antibody was obtained from Santa Cruz Biotechnology (Santa Cruz, CA, USA). The original images of immunoblot are shown in supplementary figures (Figures S7 and S8).

## 5. Conclusions

Rab8 and its modulator Rabin8 mediate EGFR signaling through the secretion of FGF in VPCs during vulval development in *C. elegans*, and this result supports crosstalk between EGFR and FGFR in the oncogenic process. In addition, this study provides conceptual support for the expansion of existing EGFR-TKI by demonstrating that the combination of EGFR-TKI and FGFR-TKI is effective against lung cancer cell lines.

## Supplementary Materials

Supplementary materials can be found at https://www.mdpi.com/1422-0067/21/20/7770/s1; Table S1: Plasmids and primers, Figure S1: RNAi screen for *30* rab genes and LET-23::GFP expression, Figure S2; Expression pattern of *rab-8*, Figure S3: Expression patterns of *rab-8* and *rabn-8*, Figure S4: Migration of sex myoblast cells in *rab-8* mutant and *jgIs25* strain, Figure S5: Quantification of proteins expressed in lung cancer cells. Figures S6–S8: Uncropped images of western blot used in figures.

## Abbreviations

EGF	epidermal growth factor
EGFR	epidermal growth factor receptor
FGF	fibroblast growth factor
FGFR	fibroblast growth factor receptor
GAP	GTPase-activating protein
GEF	Guanine nucleotide exchange facto
qRT-PCR	quantitative reverse transcription PCR
TKI	tyrosine kinase inhibitor
