# Supplementary material for: Rab8 and Rabin8-Mediated Tumor Formation by Hyperactivated EGFR Signaling via FGFR Signaling
Source: Int J Mol Sci. 2020 Oct 20;21(20):7770. doi: 10.3390/ijms21207770 (PMC7589727; doi:10.3390/ijms21207770)
Supplement: Supplementary file 1 [file ijms-21-07770-s001.pdf]

Supplementary table 1. Plasmids and primers

Plasmid	Vector	Purpose	Primer Name	Primer Sequence
pJG589	L4440	*rab-37* RNAi	W01H2.3-01	TGGATCCATGCTACTTGGTGACAGCTG
			W01H2.3-02	TATGCTAGCTCAATTAAACGTGCAACATCTGG
pJG590	L4440	*glo-1* RNAi	R07B1.12-01	TGGATCCATGGCAGCACTCACAAATAACG
			R07B1.12-02	TATGCTAGCTTAGCAACATTTCGAGTCGTATCTAG
pJG591	L4440	Y71H2AM.12 RNAi	Y71H2AM12-01	TGGATCCATGGACACTATCAAAACTGTCAAAG
			Y71H2AM12-02	TATGCTAGCCTACCATCTCTGACAGCACC
pJG595	L4440	C52B11.5 RNAi	C52B11.5-01	TGGATCCATGAGTCAAAATACAGAGTCGC
			C52B11.5-02	ATAGCTAGCTTATTCATTACTGCGAAAAAACG
pJG803	L4440	ZK970.8 RNAi	ZK970.8-02	TGGATCCGGACTTTTCATACTTGACACGTTC
			ZK970.8-03	CTTTCTAGAATGCAAGTACCACTGAAACTTC
pJG865	L4440	*mdt-9* RNAi	Y62E10A11-01	TGGATCCTGAGCAAAGAAGTGATCAAGCC
			Y62E10A11-02	TATCTCGAGTCATTTGTCTCCACTTTCACTACG
pJG635	pPD95.77	RAB-8::GFP	D1037.4-01	TATCTGCAGGATCGCATCCGGCTAGTTTATC
			D1037.4-02	AGGATCCAAGCAAATTGCAGCTCCAGTTG
pJG812	pPD95.77	EGL-17::GFP	F38G1.2-09	CTTTCCTGCAGTACATTCAATTTGAAATAC
			F38G1.2-14	TTAGGTACCGATTTTCTGGATTTCATTGTACGATAC
pJG861	pPD95.77	*rab-8p*::GFP	D1037.4-01	TATCTGCAGGATCGCATCCGGCTAGTTTATC
			D1037.4-10	ATTTCTAGAATTGGAGTGAGTGAGAGAAAAGC
pJG903	pPD95.77	*rab-8p*::GFP ::RAB-8	D1037.4-04	AGTGCTAGCTTAAAGCAAATTGCAGCTCCAG
			D1037.4-05	TAAGAATTCATGGCAAAAACTTACGACTACTTG
pJG1309	pIJ559	*F54C9.11p* ::tagRFP	F54C9.11-01	AATTGTCGACGCAGACAGTGCTTCAAAGC
			F54C9.11-02	AGGATCCCTGGGGATCGACTGACAAATC
pJG563	pPD95.77	*vha-6p*::GFP	VW02B12L1-01	TATAAGCTTCTCACGGAGATATTGCCAGCA
			VW02B12L1-02	ATAGTCGACTTATGGGTTTTGGTAGGTTTTAGTCG
pJG1076	pJG563	*vha-6*p::tdTomato ::RAB-8	D1037.4-01	TATCTGCAGGATCGCATCCGGCTAGTTTATC
			D1037.4-10	ATTTCTAGAATTGGAGTGAGTGAGAGAAAAGC
pJG1523	pJG563	*vha-6p*::GFP ::F54C9.11	F54C9.11-03	TGGATCCATGGTTGATACAATTTCTGTTCTG
			F54C9.11-04	AATGCTAGCTCAATGATGGCGTATGTTGG
pJG942	pCMV-3Tag-1A	3xFlag::RAB-8	D1037.4-03	TGGATCCATGGCAAAAACTTACGACTACTTG
			D1037.4-06	TATGTCGACTTAAAGCAAATTGCAGCTCCAGTTG
pJG1324	pEGFP-N1	F54C9.11::EGFP	F54C9.11-03	TGGATCCATGGTTGATACAATTTCTGTTCTG
			F54C9.11-06	TATGTCGACTCAATGATGGCGTATGTTGG
pJG1295	pGEX4T1	GST::F54C9.11	F54C9.11-05	TAAGAATTCATGGTTGATACAATTTCTGTTCTG
			F54C9.11-06	TATGTCGACTCAATGATGGCGTATGTTGG
pJG750	pPD95.77	*egl-15p*::GFP	F58A3.2-03	ATTTGAAGCTTGTTTTGTCCTCCTCGTG
			F58A3.2-04	CTCTCTGCAGACTGGTCAATCCTTGAAGAG

The underlined nucleotide sequence is an appended restriction enzyme site. All nucleotide sequences are listed in 5' to 3' order.

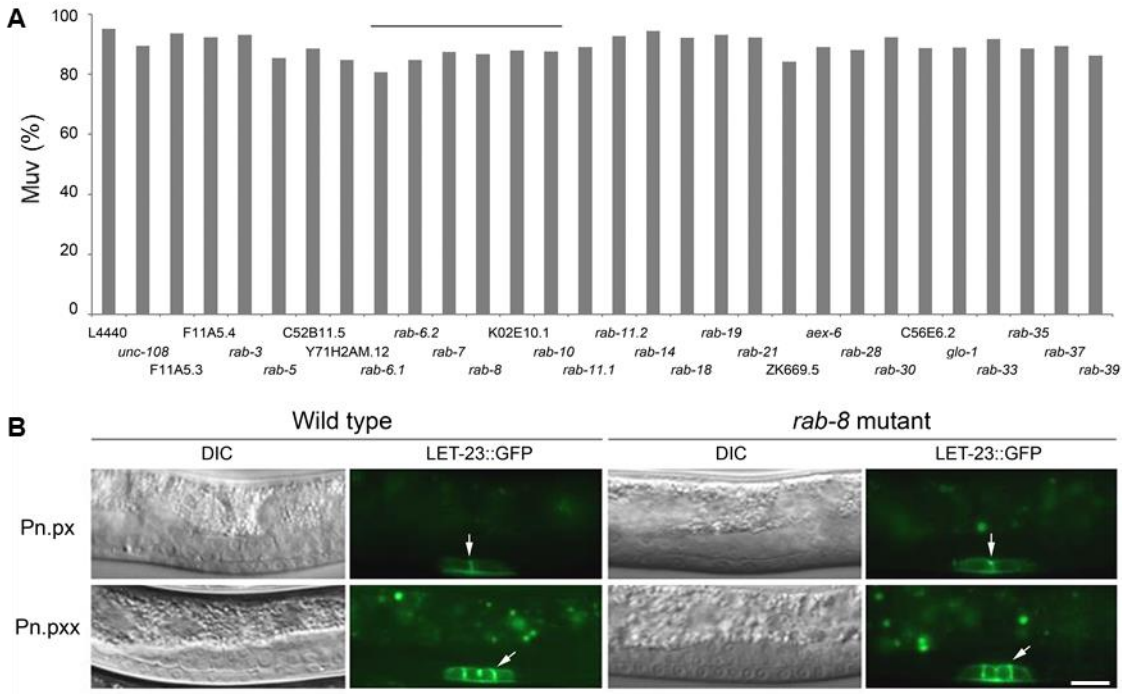

Figure S1. RNAi screen for *30* rab genes and LET-23::GFP expression. (A) The *jgIs25* strain was fed with 30 *rab* genes RNAi and worms with Muv were counted. L4440 empty vector was used as the control RNAi. The number of worms for each *rab* RNAi was counted over 100. Genes indicated by the upper line were further tested using several Muv mutants in the figure 1B. (B) LET-23/EGFR expression was examined using LET-23::GFP in the VPCs of the wild type and *rab-8* mutant. Arrows indicate LET-23::GFP in 1° VPCs of L3 larvae (Pn.px in the top panel and Pn.pxx in the bottom panel). Scale bar= 10 μm.

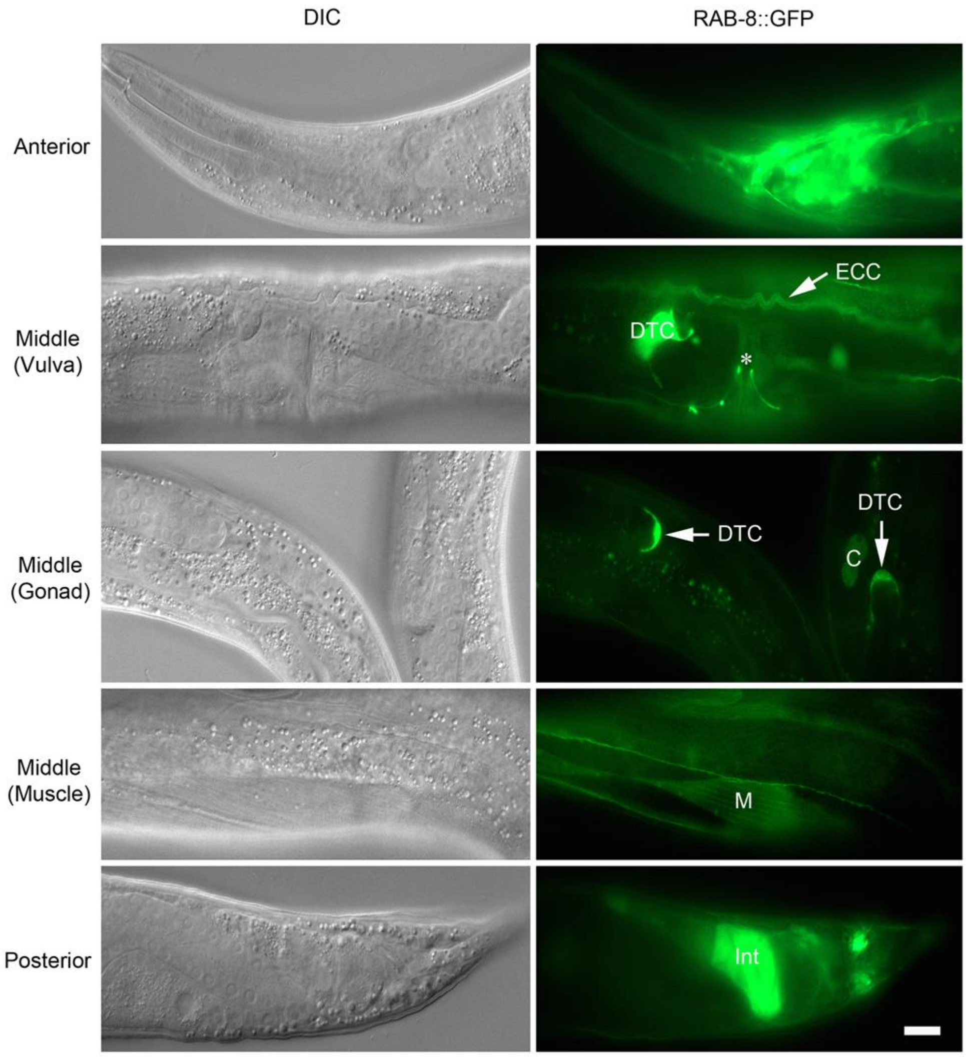

Figure S2. Expression pattern of *rab-8*. The transgenic worms expressing RAB-8::GFP were observed. Vulva (asterisk), excretory canal cell (ECC by arrow), distal tip cells (DTC indicated by arrows), coelomocytes (C), body wall muscles (M) and the intestine (Int) are indicated. Scale bar= 10 μm.

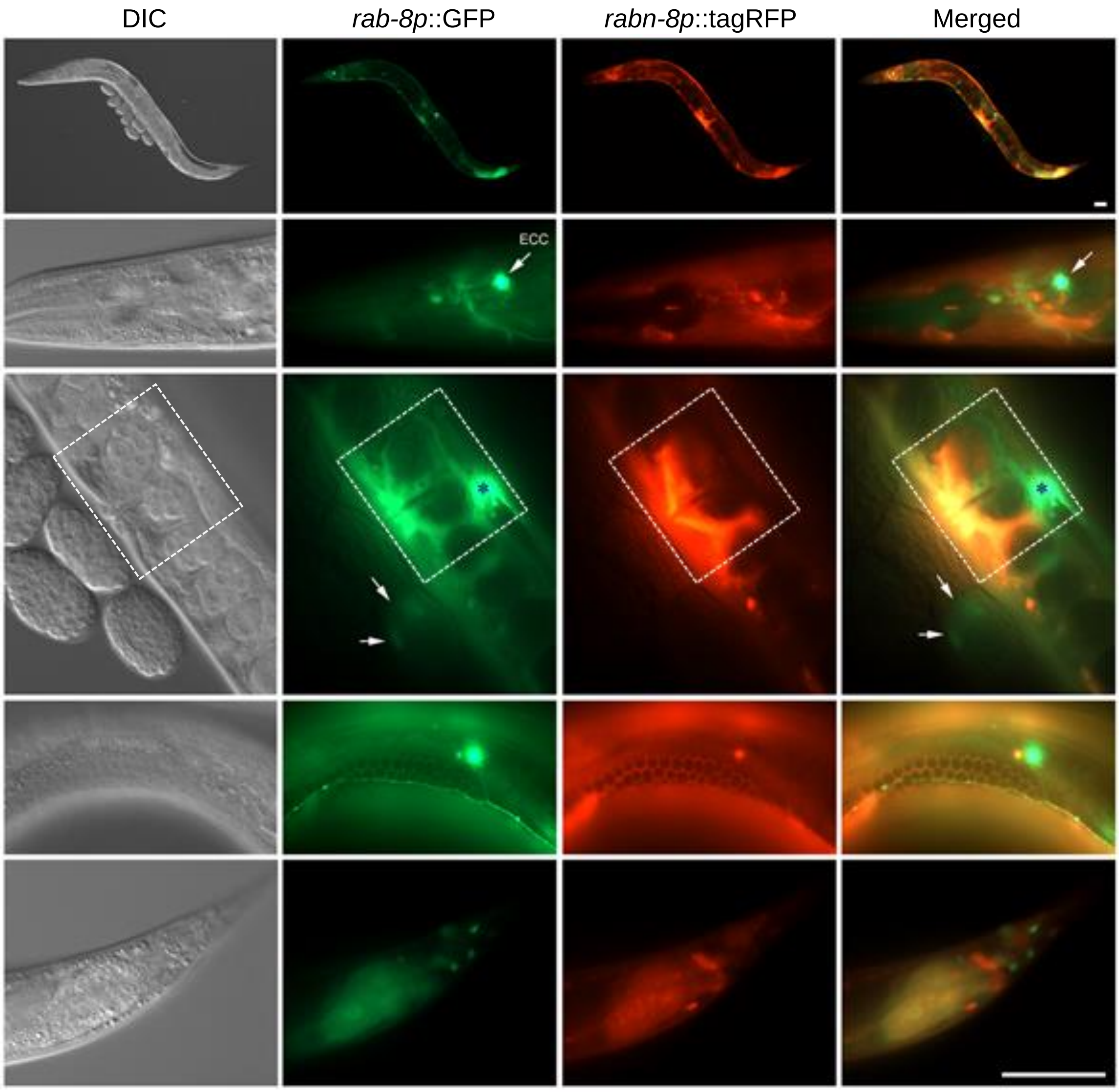

Figure S3. Expression patterns of *rab-8* and *rabn-8*. Transgenic worms that simultaneously express *rab-8p*::GFP and *rabn-8p*::tagRFP were used to investigate the expression patterns of *rab-8* and *rabn-8*. The reporter gene expresses fluorescent proteins by either the *rab-8* promoter (*rab-8p*) or the *rabn-8* promoter (*rabn-8p*). Excretory canal (ECC, arrow), distal tip cells (asterisk) and the embryo (arrows) are indicated. The boxed regions were enlarged on the figure 3F. Error bar= standard error. Scale bar= 50 μm.

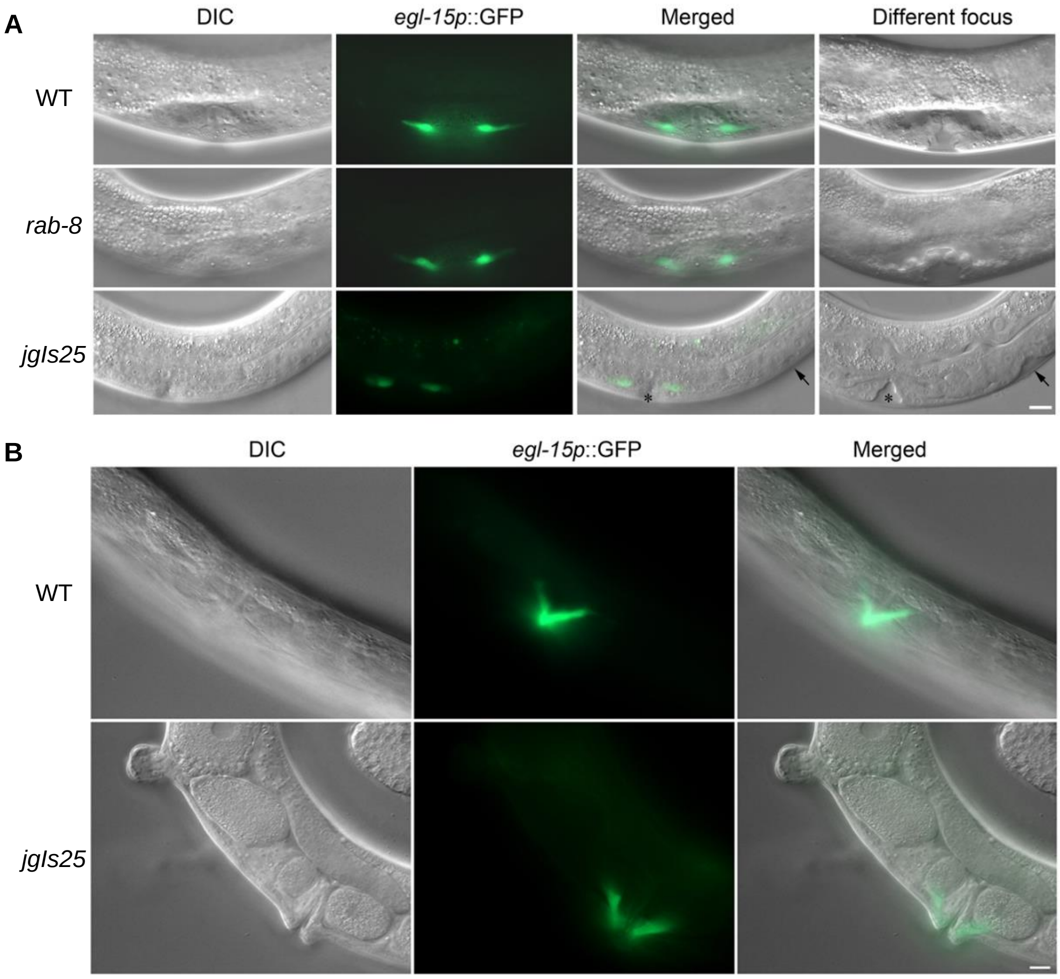

Figure S4. Migration of sex myoblast cells in *rab-8* and *jgIs25*. (A) L4 lavae expressing *egl-15p*::GFP were observed in the background of *rab-8* or *jgIs25*. Normal and extra vulva invaginations are indicated by asterisk and arrow, respectively. (B) Adult worms expressing *egl-15p*::GFP was observed in wild type or *jgIs25*. Scale bar= 10 μm.

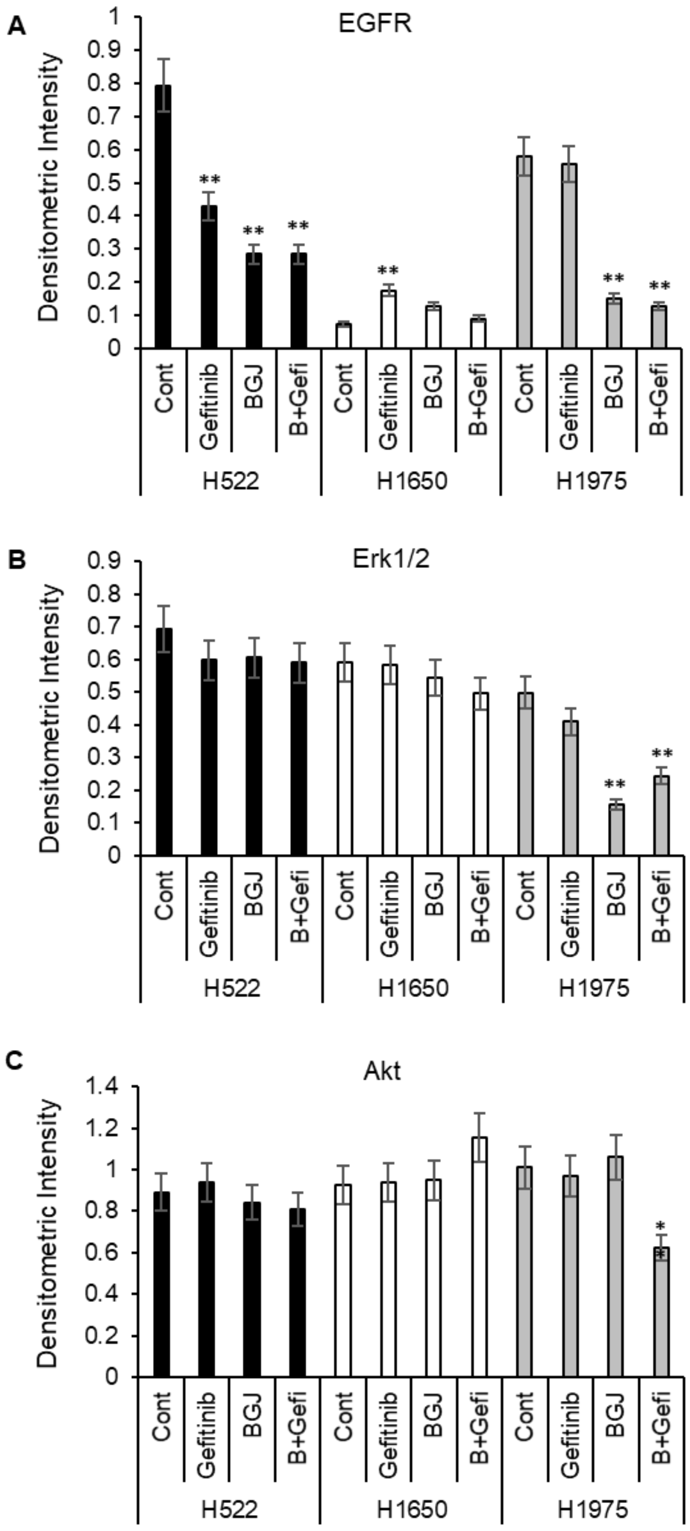

Figure S5. Quantification of proteins in figure 7D and E.

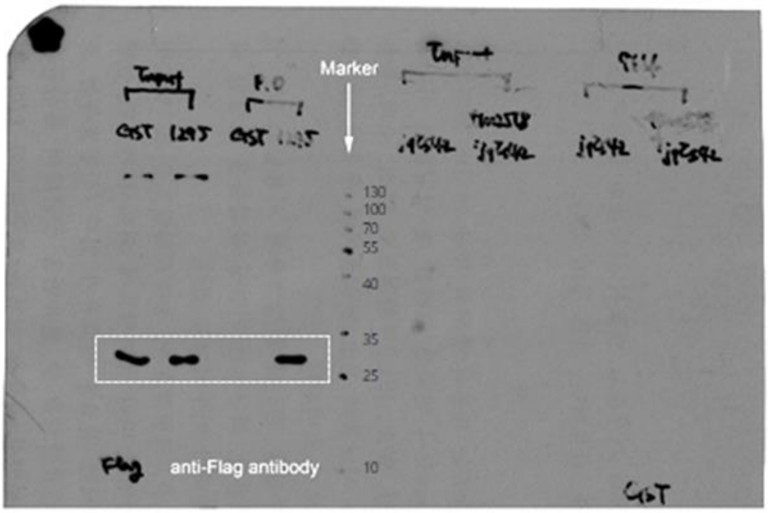

Figure S6. Original data of figure 4B.

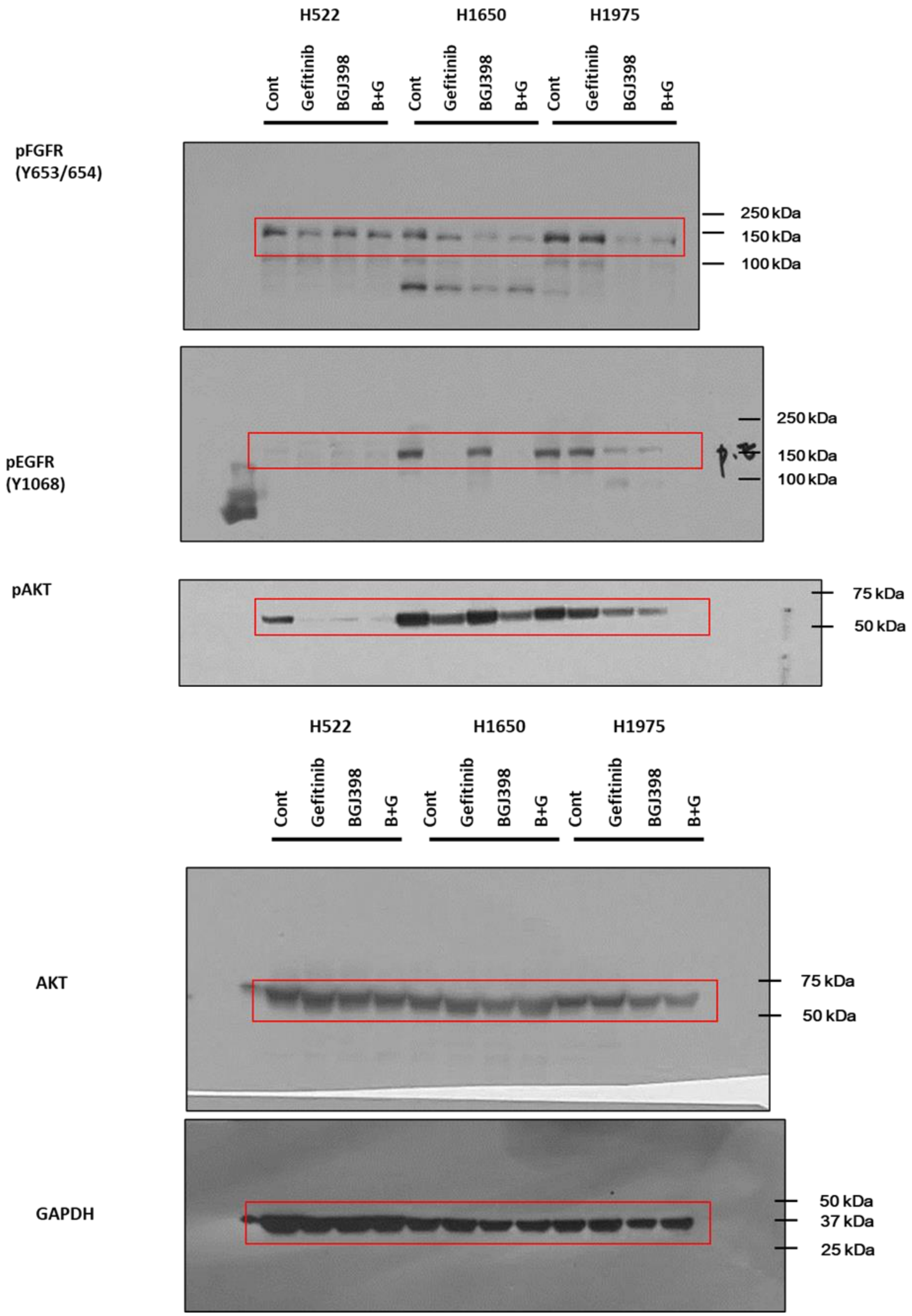

Figure S7. Original data of figure 7D.

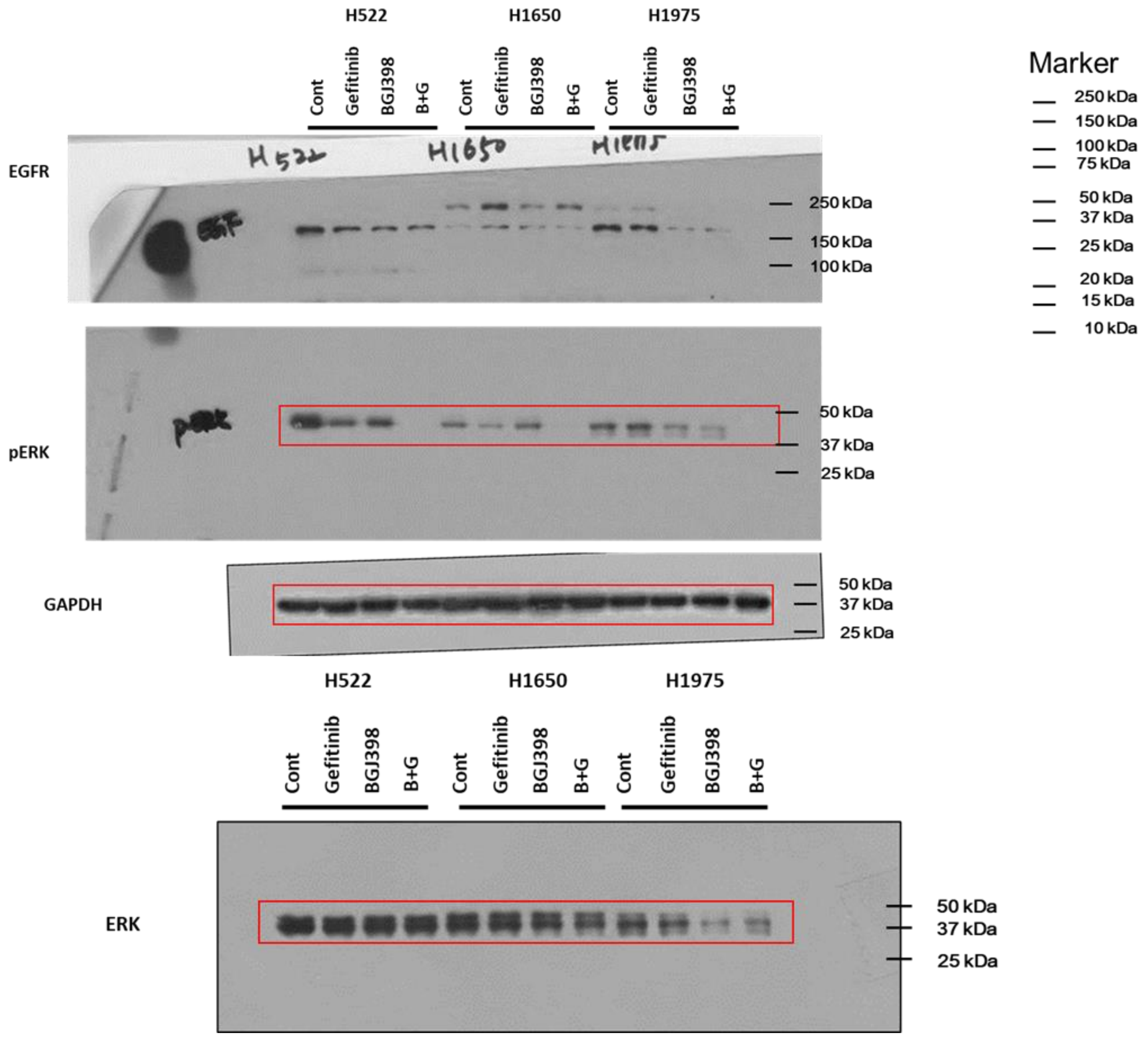

Figure S8. Original data of figure 7E.
